# Client Experiences With a Short-Term Case Management Mental Health Service

**DOI:** 10.1177/23743735221113059

**Published:** 2022-07-15

**Authors:** Andrea Duncan, Vicky Stergiopoulos, Walter P Wodchis, Maritt Kirst, Katie N Dainty

**Affiliations:** 1 Department of Occupational Science and Occupational Therapy, 7938 University of Toronto, Toronto, Ontario, Canada; 2 7978 Center for Addiction and Mental Health, Toronto, Ontario, Canada; 3 Institute of Health Policy Management and Evaluation, 7938 University of Toronto, Toronto, Ontario, Canada; 4 Implementation and Evaluation Science, Institute for Better Health, Trillium Health Partners, Mississauga, Ontario, Canada; 5 Department of Psychology, Community Psychology Program, 8431 Wilfrid Laurier University, Waterloo, Ontario, Canada; 6 Patient-Centred Outcomes, 8613 North York General Hospital, Toronto, Ontario, Canada

**Keywords:** client experience, case management, short-term case management, community, mental health

## Abstract

Short Term Case Management (STCM) was introduced in 2016 in Toronto, Ontario, as a brief intervention to address long wait-lists for case management services. STCM provides individuals with mental illness, living in the community, case management services on a weekly basis over 3 months to identify personal goals and work toward an improved state of health and well-being. Despite the small but growing body of evidence on short-term case management, there is limited research on clients’ reported experiences of these services. This study used a phenomenological approach to answer the question “What are the experiences with services of individuals who received short-term case management services?” Eight qualitative semistructured interviews were conducted between November 2019 and January 2020 to collect the perspectives and experiences of clients who had received STCM. Most participants valued engaging in a brief therapeutic relationship. Additionally, participants described that the intervention helped them connect with other agencies for ongoing support and begin achieving their own long-term goals. Some participants voiced concerns about the brief duration of the intervention. Future research should explore the role of briefcase management in the continuum of services and the typology of clients who may benefit from longer therapeutic relationship to achieve their goals.

Short Term Case Management (STCM) was introduced Toronto, Ontario, Canada, in 2016 as a briefcase management intervention to address an increasing waiting list of clients awaiting Intensive Case Management (ICM) services. Similar to ICM, STCM uses a client-centered, individualized, recovery-based approach to provide individuals with mental illness weekly case management services, identify client personal goals and needs, and work toward improving their health and well-being. The differentiating feature of STCM is that it is limited to 3 months of service. STCM was inspired by the Critical Time Intervention model, a 6 to 9-month evidence-based approach to support individuals experiencing homelessness or transitioning from institutional to community settings (1). However, STCM did not assess fidelity to a specific service model but rather focused on addressing immediate service gaps and needs.

Previous research on briefcase management found that the intervention did not reduce emergency department (ED) use or improve clients’ health and well-being (2). However, service users and providers suggest that briefcase management may improve continuity of care through a collaborative relationship (3). Despite the small but growing literature on briefcase management interventions, a better understanding of client experiences with services is instrumental to providing recovery-oriented care (4). Although we continue to evaluate clinical and psychosocial outcomes in community mental health (5), people with mental health-related disabilities have voiced that what is most meaningful for their own recovery is to have “a home, a job and a friend” (6). It is therefore essential that we listen to the perspectives of those receiving services to ensure that we are developing, operationalizing, and measuring community mental health services in an approach that is meaningful and impactful to them. This study sought to answer the question “What are the experiences with services of individuals who received short-term case management services?”

## Methods

Qualitative semistructured interviews were used to collect perspectives of clients who had received STCM services. A phenomenological approach was embraced in this research study, as the inquiry sought to understand clients’ lived experiences as told in their own words (7). This study received REB approval from the University of Toronto on September 19, 2019, with protocol # 00038074.

### Sampling and Recruitment

Clients were recruited directly through the case managers. This approach was chosen as it was perceived that this potentially vulnerable population was best recruited from within their own circle of care. Clients were eligible to participate if they had completed STCM within the past 6 months and were deemed to be able to speak to their experiential process. They were ineligible to participate if they could not provide informed consent or were deemed a safety risk. Recruitment target was set at 10 participants, or until data saturation was achieved.

### Data Collection

In-depth, one-on-one interviews were conducted between November 2019 and January 2020. All interviews were face-to-face and occurred in a location of the participants choosing, such as their home, public community space, or at the office of one of the service agencies. All clients were offered a $15 gift card as a thank you for their participation in the study. See Appendix A for the Service User Interview Guide used to facilitate the discussions. All interviews were recorded and transcribed verbatim by an external transcriptionist. Reflective field notes were taken following each of the interviews.

### Data Analysis

This study embraced hermeneutic phenomenological data analysis, in which repetitious themes were sought among the participants shared experiences and exemplar quotes were identified to highlight each theme (8). All data were entered into NVivo for analysis (9). Interviews were reread and inductive open coding was used to develop a code book. Codes were developed by one author and consensus discussions transpired within the research team. Review of field notes also occurred to challenge biases in this process. With the codebook developed 2 researchers independently deductively coded 2 interviews and then discussed and identified inconsistencies in the coding. Discrepancies were discussed until consensus was reached. Updates to the codebook were made to improve clarity and rigor. The remaining interviews were then deductively coded, analyzed by one researcher, and the data were then summarized.

## Results

After completing a total of 8 in-depth client interviews, redundancy signaled data saturation. See Table 1 for a list of clients’ pseudonyms, gender, and age. Six female and 2 male participants were included in the study, with ages ranging from 23 to 67 years and a mean age of 45.6 years. All participants reside within Toronto, ON, Canada.

**Table 1.** In-Depth Interview Participants.

Pseudonym	Gender	Age
Carrie	Female	52
Katie	Female	23
Nick	Male	41
Carol	Female	48
Donna	Female	67
Finn	Male	32
Denise	Female	66
Alison	Female	36

The themes that emerged included: (1) recovery is a shared responsibility between the client and case manager; (2) clients experienced a positive impact from the brief therapeutic relationship; (3) clients experienced challenges when the relationship was terminated; (4) clients came to STCM with a wide variety of goals and needs; and (5) clients were connected to a variety of other services and resources through the process.

### Recovery Is Shared Responsibility

Participants were asked to reflect on what we should tell future clients to expect of STCM based on their own experience. Many participants demonstrated a keen understanding that the case manager was not there to do things for them but to teach them to be resourceful.

I would say you get what you put in. You have to be as proactive to wanting results. Like don’t expect the person to do everything for you. You have to like want to change.…. They’re only as proactive as you are. So I would say if you’re serious about making the change then express and show to them that you’re serious about getting the changes done. Expect a strong support system. Or I don't know, like the way I saw it was just support. Like it’s like that helping hand to do something.—Alison, 36

This sentiment was shared among all study participants. Although some described that they initially thought the case manager would do things for them, they quickly learned that the case managers were there to guide them on their own journey.

### Positive Therapeutic Relationships

Participants often reported positive relationships with their short-term case manager and described hope and positive outcomes as a result of this therapeutic relationship.

She was kind of like a younger friend who was like, “Come on, we can do this.” …. So yeah, I was looking for a supportive friend … What I found helpful was that she actually came to my house. You know, because a lot of my issues were around how am I living like this, why am I living like this? I’m hoarding. My house is a mess. This is no way to live. So when she came in, she was kind of like, “You know what, you’re okay.” She had a picture of somebody’s…like what a hoarding situation was like. She was like, “You’re not hoarding. Like you actually have a pretty lovely place here.” And I was like I don’t feel like that. “I go into places every day, and I’m telling you, you have a pretty comfortable situation here.” So that kind of made me feel better.—Denise, 66

Although many study participants spoke of their case managers and their professional relationships, one participant described mixed views on his experience. In addition to discussing some positive experiences with the service, he expressed a desire for a more consistent relationship with his case manager.

What was bad about it was it was too… I don’t feel like it was as… Like there's no check-in. Like it would have been nice if maybe once a week, he called me or something. Or it was more like more presence. There was more like an absence between… Like an absence between situations. So like if I was to go somewhere, it was just like… Like I wouldn't hear from him for a while and then we’d be like random. You know, like it wasn’t like consistent like a week check-in in terms of just like keep updated.—Finn, 32

One participant commented that knowing that short-term case management had a limited time frame made her hold back and not feel as comfortable building as strong a relationship as she had with her psychiatrist who had been providing services for a year. Although some participants discussed that they were disappointed that the service was not ongoing, they did not share experiences of being rushed or feeling pressured during the service.

### Challenges With Ending the Therapeutic Relationship

Most participants expressed that they would have preferred to have received a long-term case management service; however, some conveyed that in retrospect they still had a positive experience with the service.

Even though it's short term, and I was kind of disappointed with that because I wanted long term because I started liking the case worker and, you know, the rapport was great. … And just leaving here knowing that somebody actually cares, that actually genuinely cares and wants to help made a huge difference … Once I left here, that negative thinking went out the window. So short term case management can actually help people get away from negative thinking about case managers and organizations. Because you hear a lot of horror stories out there about case managers and organizations. So it does help restore your faith in humanity.—Nick, 41

One participant, in particular, made it clear that she was not happy that the professional relationship had come to an end. During the interview, she regularly returned to the concept that the loss of the case manager was a difficult experience for her.

If someone’s mental health is messed up, whether it’s because they have…they’ve always had issues or because now they’re not well, to plant someone into their world who they get comfortable with because it takes time to get comfortable, and then to take that away… I mean eventually I guess you have to move on. It’s just the way life works. But I think that if it can’t be you then it needs to be someone else just to visit, just a simple visit. One hour a week or one hour every other week makes such a big difference. Such a big difference. It took me a few weeks to adjust back to no one to talk to. You know what I mean, I have family. It’s not like I don’t. …. I mean I miss her. I think about her all the time. As silly as that sounds. We didn’t know each other very long but I felt like we made a connection.—Donna, 67

And another participant described similar views.

But it’s short term. So it’s almost like… It’s kind of like if you have your mother with you the whole time and then all of a sudden your mother just, you know, says, “Hey, you’re on your own.” And then it’s kind of like that.—Finn, 32

### Goals and Expectations

This research sought to examine how STCM can address client personal recovery goals. A few participants articulated that they were not sure what to expect from STCM and did not start the process with any particular goals in mind. Some articulated that they were quite sick at the start and were not able to articulate their own goals. However, most participants discussed that the “work” with the case manager helped them identify and articulate appropriate goals for service.

Okay. I joined the short-term counselling … not really knowing what to expect and not really knowing what I needed, especially because I was going through an emotional crisis. So, I was a little confused as to what I wanted and what I expected … she gave me time to explain my situation. She listened carefully. She told me the length of the counselling and what it could offer. And then between the first and second interview, I was able to collect my thoughts and realize what resources I wanted to access …. And it went on from there.—Carol, 48

Conversely, some participants came to the case management experience with very clear goals for STCM.

My main goal short term was to get more on my feet and to be able to find employment and get access to different social services.—Katie, 23

Further to this, a few participants were very clear that their goals were focused on symptom management and reducing their psychological distress.

My goal is try to reduce, maybe it’s a good word, my anxiety.—Carrie, 52

Well, I guess my goals at that time were just to really stop the discomfort. … Not so much physical but emotional discomfort and, you know, all those things. Feeling guilty, cowardice and shame. Just like really difficult emotions, right. So I was more trying to find somebody… Like I was trying to find a professional friend. You know, somebody who would kind of encourage me and help me sort things out.—Denise, 66

### Supporting Connection to Other Services

The most consistent theme emerging from participant narratives was that the services were in line with a broker case management model. Participants commonly described that their case manager connected them with a variety of services, agencies, and resources to address their needs. Most participants did not express concern that the case manager was not with them until their goal was achieved and expressed appreciation that they had at least been connected with other service agencies.

And then I was really overwhelmed with just basic stuff. Like I needed new glasses, I needed new teeth. So, she helped me just kind of organize all that stuff. Like I found I really couldn't fill out forms, I couldn't call. You know, I could maybe find the place on the Internet okay. But then I’d go to call, and they’d put you on hold or… You know, she knows how to do that. She’s doing that all day long. … She’d bring the form, fill everything out. And sure enough, stuff started happening. She took me to get glasses. She found a program where I can get them for free. … So practical things like that. She got me on a waiting list for dental services with the city.—Denise, 66

## Discussion

This study sought to understand clients’ experiences with a Short-Term Case Management intervention using qualitative methods. Clients consistently described that participating in a recovery-oriented service meant that they were responsible for contributing to the case management process and did not expect their case manager to do everything for them. Additionally, clients valued the therapeutic relationship with a case manager, even if brief. Although most participants described positive experiences with the therapeutic relationship, some conveyed concerns that this relationship was time limited or voiced a desire to interact more frequently with their case manager.

Our findings support and are supported by previous research on the importance of creating “partnership” with clients to support positive health and well-being outcomes (10). Further to this, the partnership itself is important to engaging clients in the recovery process (11) through the therapeutic relationship. It was not surprising then that clients in this study often discussed the relationship with their case manager as the most impactful aspect of their STCM experience. The clients specifically shared stories of “feeling listened to” or “being seen” or “feeling validated.” This was highlighted as being impactful to supporting clients achieve their goals. This meaningful interaction and dialogue were described as a positive and therapeutic way to help clients feel engaged in their own goal achievement. Additionally, some clients highlighted that this relationship had elements of a friendship. This notion is reflected in other studies of briefcase management in which clients have expressed views that a positive aspect of a briefcase management experience is to have a “friendly” case manager who leverages a recovery approach (3). Additionally, this study mirrors other findings that highlight that across most community mental health interventions, it is the therapeutic working alliance that is most linked to improved outcomes (12).

Similarly, some clients conveyed concern about the “termination” of this therapeutic relationship. This sentiment is not unique to clients completing an STCM intervention. It is known that discharge is an especially risky time for clients receiving mental health services as it can lead to relapse or worsening of symptoms (13). Other studies have also reported that clients experience feelings of abandonment when services are terminated (14,15). This key construct presents an especially complex issue for community mental health service provision. Although some clients expressed concern over termination of case management services, continued service has the potential to promote dependency on the therapeutic relationship thus making future discharge even more difficult. Case management services need to manage wait-lists and serve those most in need, discharging clients where appropriate, even when clients express concern or dissatisfaction with the ending of the relationship.

### Limitations

This study’s main limitation is recruitment bias. Case managers supported the identification of potential participants for the study, and therefore it is likely that this study spoke only to clients who had had a positive experience with STCM. As a result, findings may reflect the clients’ perspectives and experiences of STCM when it works well. Nonetheless, not all experiences elicited were positive, and some concerns did emerge in the data. The study may also have more heavily recruited participants who had more insights to contribute, who may not be representative of the bigger sample. Every effort was made however to make research accessible, including the location of the interview and tailoring the interview guide to the needs of individuals with serious mental illness.

## Conclusion

This study described client experiences with STCM services. Most study participants described benefits from engaging in STCM and appreciated a brief therapeutic relationship. Future research should examine the role of STCM in service delivery and the typology of clients that might benefit from a longer therapeutic relationship.

## Supplemental Material

sj-docx-1-jpx-10.1177_23743735221113059 - Supplemental material for Client Experiences With a Short-Term Case Management Mental Health Service Click here for additional data file. Supplemental material, sj-docx-1-jpx-10.1177_23743735221113059 for Client Experiences With a Short-Term Case Management Mental Health Service by Andrea Duncan, Vicky Stergiopoulos, Walter P Wodchis, Maritt Kirst and Katie N Dainty in Journal of Patient Experience
