# Supplementary material for: Client Experiences With a Short-Term Case Management Mental Health Service
Source: J Patient Exp. 2022 Jul 15;9:23743735221113059. doi: 10.1177/23743735221113059 (PMC9289903; doi:10.1177/23743735221113059)
Supplement: sj-docx-1-jpx-10.1177_23743735221113059 - Supplemental material for Client Experiences With a Short-Term Case Management Mental Health Service [file sj-docx-1-jpx-10.1177_23743735221113059.docx]

**Appendix A – Service User Interview Guide**

1. Can you please share your experience with short term case management services?

Probing Questions

- 1. What are your goals for case management services?
  2. What were your most significant needs when starting out with this service?
  3. What steps has your case manager taken to help you address these needs?
  4. Have any steps along the journey been more helpful? Have any been less helpful?
  5. What has been the most effective thing your case manager has done to help you achieve your goals?

1. What do you think are realistic and achievable outcomes for your short-term case management experience?

Probing Questions

- 1. What do clients need to hear to help set their expectations?
  2. What goals do you think clients are able to achieve in a limited time frame?
  3. What goals are not achievable in short term case management?
  4. Why is this?

Now I would like to share some findings with you. We looked at the OCAN data from 2016 – 2018. We looked at service user and service provider assessment of need. We also looked at the initial and discharge findings.

1. What is your initial reaction to this data?

Probing Questions

- 1. Is this information surprising, or is it what you would expect to see?
  2. If it is not what you would expect, why do you think this is?
  3. What is missing from this data?

1. Now that you have seen this, I’d like to ask you a few questions again to see if your opinions have changed or remain the same?
   1. What do clients need to hear to help set their expectations?
   2. What goals do you think clients are able to achieve in a limited time frame?
   3. What goals are not achievable in short term case management?
2. Is there anything further you would like to share with me about your experience with Short-Term Case Management?

Thank you for your time today.
